# Thyroid Hormone Action: Astrocyte–Neuron Communication

**DOI:** 10.3389/fendo.2014.00082

**Published:** 2014-05-30

**Authors:** Beatriz Morte, Juan Bernal

**Affiliations:** ^1^Instituto de Investigaciones Biomédicas “Alberto Sols”, Consejo Superior de Investigaciones Científicas, Center for Biomedical Research on Rare Diseases (CIBERER), Universidad Autónoma de Madrid, Madrid, Spain

**Keywords:** thyroid hormone, type 2 deiodinase, astrocytes, fetal and postnatal brain, thyroid hormone transporters, T3 availability

## Abstract

Thyroid hormone (TH) action is exerted mainly through regulation of gene expression by binding of T3 to the nuclear receptors. T4 plays an important role as a source of intracellular T3 in the central nervous system via the action of the type 2 deiodinase (D2), expressed in the astrocytes. A model of T3 availability to neural cells has been proposed and validated. The model contemplates that brain T3 has a double origin: a fraction is available directly from the circulation, and another is produced locally from T4 in the astrocytes by D2. The fetal brain depends almost entirely on the T3 generated locally. The contribution of systemic T3 increases subsequently during development to account for approximately 50% of total brain T3 in the late postnatal and adult stages. In this article, we review the experimental data in support of this model, and how the factors affecting T3 availability in the brain, such as deiodinases and transporters, play a decisive role in modulating local TH action during development.

## The Model

Thyroid hormone (TH) action is exerted mainly through regulation of gene expression by binding of T3 to the nuclear receptors (1, 2). T4 plays an important role as a source of intracellular T3 in the central nervous system via the action of the type 2 deiodinase (D2). This process is regulated physiologically. D2 activity in brain increases during development in correlation with the more T3 sensitive developmental period. D2 activity and T3 concentrations are synchronized in a spatial and temporal fashion to determine critical brain processes such as myelination, neuronal migration, glial differentiation, and neurogenesis. On the other hand, D2 activity is inversely regulated by T4, so that brain T3 levels fluctuates less than circulating TH levels, due to changes in D2 activity in response to changes in T4 availability at least at postnatal and adult stages (3).

Although neurons are the primary target of T3 actions, Guadaño-Ferraz et al. (4) demonstrated that D2 expression takes place predominantly, if not exclusively, in glial cells: the tanycytes (5) lining part of the third ventricle surface and in the astrocytes throughout the brain. The *Dio2* mRNA was not restricted to the cell body but was also present along the cellular processes. This observation indicated an important role for glial cells in TH homeostasis in the brain and a close coupling between glial cells and neurons in TH metabolism. According to these observations, the authors suggested a model of T3 availability to neural cells. On the one hand, circulating T4 and T3 would enter the brain through the blood–brain barrier (BBB). T4, upon entering the brain would reach the astrocytes through their end-feet in contact with the capillaries, and produce additional T3 by D2-mediated deiodination.

## Evidence from Transcriptomic Data

Support for the glial specificity of Dio2 expression came from transcriptomic studies by Cahoy et al. (6). These authors performed transcriptomic studies in primary neural cells isolated from the mouse brain, without further manipulations to establish the patterns of gene expression representative from the different cell types *in vivo*. *Dio2* was enriched up to 50 times in the astrocytes, and therefore may be considered as a highly specific astrocyte gene. Despite this cellular specificity, there is evidence that *Dio2* is also expressed in cells other than the astrocytes in some situations. For example, in profound hypothyroidism in the rat, *Dio2* expression was also observed in a fraction of cerebral cortex interneurons (7). It was more recently demonstrated that astrocyte-specific inactivation of *Dio2* (GFAP–Cre–D2KO mice) reduced D2 activity in brain to less than 10% of the control (8).

## Evidence from Paracrine Interactions *in vitro*

Freitas et al. (9) studied *in vitro* the evidence for a paracrine interaction between astrocytes and neurons. The goal was to check whether the T3 generated in glial cells was able to activate neuronal gene expression. This experimental system was based on an *in vitro* co-culture system of H4 human glioma cells expressing D2 and neuroblastoma cells. The two cell types were located in two adjacent compartments bathed with the same culture media. The authors demonstrated that upon incubation with T4, D2 activity in glial cells resulted in increased T3 production that reached neurons and promoted TH-responsive gene expression.

## Evidence from Transporter Pathophysiology: Direct Functional Demonstration of the Paracrine Model *in vivo*

A large body of evidence has accumulated in recent years demonstrating the crucial importance of transporters in mediating the cellular uptake of THs through the cell membranes (10). The most specific and physiologically relevant transporters for THs identified so far are the monocarboxylate transporter 8 (MCT8, SLC16A2), and the organic anion transporting polypeptide 1C1 (OATP1C1, SLCO1C1). MCT8 mutations cause an X-linked syndrome with severe psychomotor retardation and elevated serum T3 levels, indicating the importance of this transporter in TH availability to the brain (11, 12). Other transporters may also contribute to this process although their specific roles are less clear.

MCT8 transports T4 and T3 (13) and OATP1C1 exhibits a remarkable affinity and specificity toward T4 and rT3 (14, 15). Roberts et al. (16) reported the presence of Oatp1c1 protein in the abluminal side of endothelial capillary cells forming the BBB. The Oatp1c1 signal overlapped partially with aquaporin 4, a marker of astrocytes’ end-feet, which are in contact with brain micro capillaries. Mct8/MCT8 is also expressed in the micro capillaries in rodent and human, but not in the astrocytes’ end-feet. In addition, Mct8 is also expressed in neurons and choroid plexus (17).

Based on the preferences for TH transport and on the expression patterns of these two transporters, delivery of circulating T4 and T3 to the brain may be formulated in the following way: circulating T3 and T4, crossing the BBB through Mct8 would be delivered to the extracellular fluid, reaching directly the neural cells in the proximity of the micro capillaries. On the other hand, T4, but not T3, would be delivered directly to the astrocytes after Oatp1c1-mediated transport through the BBB. It is important to notice that OATP1C1 is poorly expressed in the BBB of human fetus (16) and adult primates (18) and therefore in the human brain, T4 transport is dependent on MCT8.

We provided experimental evidence in support for this model in rodents by measuring the relative effects of T4 and T3 on brain gene expression in *Mct8* knockout mice (Mct8KO) (19). T3 or T4 were administered to wild-type (WT) and to Mct8KO mice previously made hypothyroid, and expression of two neuronal target genes was measured in the cerebellum and striatum. Whereas T4 and T3 were similarly active in WT mice, the Mct8KO mice only responded to T4. The data suggested that the critical restriction to T3 transport in the absence of Mct8 is located at the BBB rather than at the plasma membrane of individual neurons, where other transporters can substitute for Mct8. The similarity in the effects of T4 in the Mct8-deficient and in the WT mice, suggested that T4 can reach the astrocytes in the Mct8-deficient mice through a different transporter, most probably Oatp1c1, and produce enough T3 to regulate neuronal gene expression through a paracrine interaction. Actually, the increased D2 activity present in the Mct8KO mice (20, 21), would facilitate this pathway in face of lower circulating T4, and restricted brain uptake of T3. The consequence is that the Mct8KO mice maintain brain gene expression similar to WT mice, with a few exceptions. Direct proof that D2 activity was indeed responsible for normal expression of most brain TH-regulated genes in the absence of Mct8 was also provided (22). In the Mct8KO mice, inactivation of D2 led to patterns of brain gene expression that were similar to that of severely hypothyroid mice, highlighting the importance of D2.

Therefore, the studies on the Mct8-deficient mice evidenced that the factors affecting T3 availability in the brain, namely deiodinases and transporters, play a decisive role in modulating local TH action. These studies also gave a functional direct demonstration of the critical role of astrocytes in regulating the amount of T3 available for neuronal uptake *in vivo*. The model also explains why the double inactivation of Mct8 and Oatp1c1 transporters (23) leads to a situation in the brain similar to hypothyroidism, as in the double Mct8 and Dio2 KO.

## The Relative Contributions of Direct T3 Uptake from the Circulation and the Local T3 Production in the Astrocytes

### Postnatal and adult rodents

**Figure 1:**
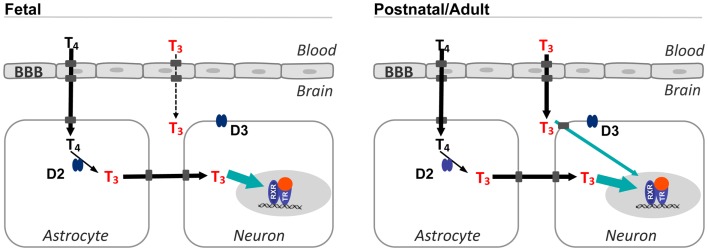
**Sources of T3 in the fetal and postnatal brain**. T4 from the circulation crosses the BBB and reaches the astrocytes and is converted to T3 by type 2 deiodinase. This is the main source of T3 in the fetal brain. In postnatal and adult animals, T3 from the circulation can also access the brain. The proportion of T3 from the circulation increases up to 50% in the late postnatal stages. *In situ* T3 production accounts for a high occupancy of thyroid hormone receptors (green arrow), and is also important for expression of genes regulated negatively by thyroid hormone. Transporters in the cell membranes are represented by gray squares, without naming any specific transporter. Although in the rodent brain, the BBB expresses Mct8 and Oatp1c1, the latter is not present in the primate BBB. T4 enters the astrocytes most likely through Oatp1c1. Passage of T3 from astrocytes to neurons is facilitated by Mct8 and also other transporters, since in Mct8 KO mice there is no apparent restriction for the passage of astrocytic T3 to neurons. Type 3 deiodinase is localized in the plasma membrane of neurons. BBB, blood–brain barrier; D2, type 2 deiodinase; D3, type 3 deiodinase; TR, thyroid hormone receptor; RXR, 9-cis retinoic acid receptor.

In the D2-deficient mice, brain T3 concentrations at postnatal day 15 are about half of normal, suggesting that at least from this postnatal age onward, 50% of the total brain T3 derives from direct T3 uptake from the circulation and another 50% derives from local T3 production in the astrocytes (24) (Figure 1). In support of this, Mct8 inactivation also leads to roughly 50% reduction of brain T3 (21).

Local production of T3 accounts for a much higher occupancy of brain nuclear receptors compared to the liver. Crantz et al. (25) estimated that local conversion of T4 to T3 accounted for 77% of the nuclear T3 receptor occupancy in the cerebral cortex and up to 37% in the cerebellum. Circulating T3 was responsible for about 20–25% of nuclear receptor occupancy. These authors concluded that the nuclear receptors were almost saturated by T3 in the cortex, and at 60% of total receptor capacity in the cerebellum. Full receptor saturation under euthyroid conditions may explain why TH-responsive genes in the cortex of euthyroid animals do not respond or do so modestly above the normal expression level after administration of excess T3. In studies of T3 administration to *Dio3* KO mice, we found that the response to high doses of T3 was higher in D3-deficient mice than in the WT euthyroid mice (26). Therefore, nuclear occupancy was not a limiting factor in gene responses to T3, and the reason why the euthyroid brain does not respond genomically to excess T3 is due to the protective action of D3.

Despite the importance of D2 for the local generation of T3, D2-deficient mice showed normal expression of several genes positively regulated by T3 (24). We confirmed these observations for additional genes, and showed that in the absence of D2, the expression levels of positively regulated genes are maintained by T3 from the circulation (22). However, expression of the negatively regulated genes is more frequently affected by D2 inactivation, suggesting that they are sensitive to the source of T3. The mechanism underlying these observations is unknown, and somehow the source of T3 may influence directly or indirectly the cellular T3 content.

### Thyroid hormone transport in the fetal brain

The fetal brain depends almost entirely from T4 monodeiodination, so that most brain T3 is produced locally from T4 (Figure 1). In the human fetal brain, despite the presence of T3 receptors and T4 in tissues by the second trimester, and even before, only the brain showed a significant T3 concentration in the nucleus as compared with other tissues (27). This observation indicates that the main source of T3 in the fetal brain is local T4 deiodination. In support of this idea, D2 activity rises in the human cerebral cortex during the second trimester in parallel with T3 concentrations (28).

In the rat (29, 30), physiological doses of T4 administered to hypothyroid pregnant rats could normalize T3 concentrations in the brain and increase neuronal gene expression. In contrast, administration of even large doses of T3 to the mother failed to increase T3 concentration in the fetal brain despite reaching other tissues, and was not able to normalize fetal gene expression. The rat fetal brain contains significant D2 activity (31) making it plausible that most if not all brain T3 is produced locally.

## Why Does Circulating T3 Cannot Enter the Fetal Brain?

The reason why the fetal brain is apparently impermeable to circulating T3 is unknown. A possible explanation could be a lack of expression of the Mct8 transporter. To address this issue, the expression of Mct8 and of the specific T4 transporter Oatp1c1 were analyzed by confocal microscopy in the prenatal rat cortex. Both proteins were present in the brain capillaries and in the epithelial cells of the choroid plexus (30).

If T3 cannot enter the brain despite expression of Mct8, what is the role of this transporter in the fetal brain? It is possible that Mct8 at early stages of development has a more prominent role in TH efflux from the brain (32, 33) as proposed in the liver for Mct8 and Mct10 (34). Another possibility is that circulating T3 crossing the BBB through Mct8-mediated transport is deposited directly in the extracellular fluid where it could be rapidly degraded by neuronal D3 activity (35). D3 is abundantly expressed in fetal tissues including the brain (36, 37), where it is expressed in neurons (38, 39) and serves as a fine regulator of tissue TH concentrations. For example, in the human fetal brain, D3 activity in the cerebellum prevents the accumulation of T3 during mid-gestation (28), at the same time that D2 activity is responsible for T3 accumulation in the cortex. Hernandez et al. (40) demonstrated that D3 plays a critical role in maintaining low levels of TH during fetal and early neonatal life in mice. During postnatal development, disruption of the *Dio3* gene increases basal expression of the T3-target gene *Hr* (41). The catalytic site of D3 was earlier proposed to face the extracellular fluid (42, 43). If this was true, T3 in the extracellular fluid of the fetal brain would be easily accessible to D3-mediated degradation. However, more recent evidence derived from *Dio3* transfection experiments indicates that D3 substrates need to be internalized for degradation (44). Even so, it may be speculated that the topography of D3 localization in the plasma membrane could allow easier and faster degradation of substrates crossing the neuronal membrane from the extracellular fluid. Within this context, one attractive, but still speculative hypothesis is that the increased proportion of T3 entering the brain from the circulation, taking place from fetal to postnatal stages, is due a decrease of D3 activity. The contribution of systemic T3 would then increase in parallel to account for approximately 50% of the total brain T3.

## Local Generation of T3 from T4 in the Context of Astroglial Maturation

The contention that in the fetal brain most T3 derives from T4 has to be examined in the context of D2 activity and maturation of D2-expressing astrocytes during development. The main surge of D2 activity in the rat brain is postnatal, with a peak around postnatal day 15, a time in which the highest T3 concentration in the brain is reached. In the fetal brain, D2 activity is low, and shows a discrete peak just before birth, at prenatal days 18–21 (31, 45). Consequently, brain T3 concentrations during the perinatal period are low, about half to one-third of adult rats.

Some aspects of the model are not well-understood, and should be refined to take into account several factors. One is astrocyte development, which is mainly postnatal (46), increasing in number during postnatal stages, and following a pattern similar to D2 activity. Although the role of astrocytes during the fetal and early postnatal periods has been questioned (47), native astrocytes isolated from the P1 mouse brain already contain a high concentration of *Dio2* mRNA (6). It may be that the small population of astrocytes present in the last few days of fetal stages has an important role in the local formation of T3 in the brain. Another difficulty is to extrapolate the model to the human situation. The human brain expresses little OATP1C1 in the BBB, and T4 and T3 transport apparently rely exclusively on MCT8. Therefore, entry of T4 to the astrocytes has to take place through a different transporter. Specifically addressing the patterns of thyroid hormone transporter, and deiodinases in the fetal and postnatal human brain should shed light on these issues.

## Conflict of Interest Statement

The authors declare that the research was conducted in the absence of any commercial or financial relationships that could be construed as a potential conflict of interest.
